# Ghosts from the Nursery: Attachment, Adverse Childhood Experiences, and Maternal Mental Health

**DOI:** 10.1007/s40653-025-00803-0

**Published:** 2026-01-09

**Authors:** Maria Balaceanu, Amanda Ramos, Mattison Hillin, Ives Hong, Dannelle Larsen-Rife

**Affiliations:** 1 Department of Human Development and Family Studies, Utah State University, Old Main Hill, Logan, UT 84322 USA; 2 Department of Psychology, Utah Tech University, 225 S University Avenue, UT 84770 St. George, USA

**Keywords:** Insecure attachment, Maternal mental health, ACEs, Anxiety, Avoidance

## Abstract

**Background:**

Children’s attachment is shaped by their mother’s mental health and their early life experiences. Motherhood evokes vulnerability, anxiety, anger, and helplessness. Poor maternal mental health may impede mothers from responding to their children’s needs.

**Objective:**

The study aimed to examine the influence of maternal mental health and adverse childhood experiences (ACEs) on adult attachment anxiety and avoidance.

**Participants and Setting:**

Adult participants (*n* = 317) were recruited through a small university in the southwest United States via social media, class announcements, and word of mouth on campus and in the surrounding community.

**Methods:**

Participants completed online survey questions assessing their perceptions of their mothers’ mental health during childhood, ACEs, and adult attachment anxiety and avoidance. Hierarchical regressions were employed to evaluate the effects of ACEs and maternal mental health on predicting attachment anxiety and avoidance.

**Results:**

The presence of ACEs showed a significant positive association with attachment anxiety and avoidance. Furthermore, the findings indicated poor maternal mental health during childhood was linked to higher levels of anxiety and avoidance in adulthood, over and above the presence of ACEs alone.

**Conclusions:**

Maternal mental health combined with ACEs is a stronger predictor of insecure adult attachment than ACEs alone. These results underscore the importance of addressing maternal mental health in interventions aimed at promoting secure attachment in children. Early identification and support for mothers experiencing mental health challenges may mitigate the risk of insecure attachment in their children, thereby promoting healthy child development, with benefits that continue into adulthood.

## Introduction

Attachment is a fundamental aspect of human behavior “from the cradle to the grave” (Bowlby 1979). It begins in infancy, with the primary goal being to establish a secure emotional bond with the primary caregiver, typically the mother. This early connection forms the foundation for future relationships (Hazan and Shaver 1994; Karr-Morse & Wiley 2013); Lyons-Ruth 2002). When mothers are unable to provide adequate care and affection, often due to poor mental health, children may have difficulty forming close emotional and physical connections later in life (Atif et al. 2015; Barone and Carone 2021; Khan and Renk 2019; Piché et al. 2011). Confidence in a consistently responsive primary caregiver allows the infant to explore and develop optimally, fostering a lifelong belief in secure, trusting relationships (Ainsworth 1969); Bowlby 1969, 1979; Hazan and Shaver 1994; Lyons-Ruth 2002). In addition to maternal mental health, adverse childhood experiences (ACEs) also significantly impact child development and shape attachment styles in adulthood (Anda et al. 2005; Perry 1999). Children who experience ACEs often have difficulty with self-regulation and may also show an exaggerated perception of threat and a chronic fight-or-flight state (Field et al. 1988; Perry 1999; Raby et al. 2017; Scully et al. 2020). Risk increases in a dose–response manner, particularly for those with four or more ACEs (Webster 2022). This overactivation of the stress response system may undermine trust in others and impede the development of skills needed to form meaningful, secure relationships with family, friends, and later romantic partners (Hazan and Shaver 1994).

Although early experiences were long underestimated due to the misconception young children would not remember them, research over the past few decades has increasingly highlighted their critical role in shaping future relationships and attachment styles (Anda et al. 2005; Felitti et al. 1998; Scully et al. 2020).

### Attachment Theory

During the early years of life, children’s primary goal is to determine if they can rely on their primary caregiver in times of need for safety and security (Bowlby 1969). According to attachment theory, this assessment results in one of three responses:*yes*,* no*,* or maybe *(Ainsworth 1969); Bowlby 1969; Hazan and Shaver 1994; Lyons-Ruth 2002). Based on the consistency and quality of their primary caregivers’ responses, children adapt their behaviors accordingly. Children met with inconsistent or intrusive care typically develop attachment anxiety (Bowlby 1969), while consistently unresponsive care is associated with attachment avoidance (Bowlby 1969). In contrast, children typically form secure attachments when their caregivers are consistently responsive to their needs (Bowlby 1969). The quality of caregiver responses shapes the emotional and psychological development of children into adulthood (Hazan and Shaver 1994; Lyons-Ruth 2002). In early childhood, attachments are considered complementary, with the primary caregiver, typically the mother, providing all the care, while the child, unable to reciprocate, receives this care (Bowlby 1969; Hazan and Shaver 1994; Lyons-Ruth 2002). In contrast, adult attachments are typically reciprocal, with both individuals giving and receiving care (Hazan and Shaver 1994). Early attachment to a primary caregiver often persists into adulthood, and patterns of anxiety or avoidance established in childhood continue to influence adult relationships, whether in ongoing relationships with parents or in romantic partnerships (Erickson et al. 2019; Hazan and Shaver 1994; Lyons-Ruth 2002; Raby et al. 2017; Ruiz et al. 2018). For example, Zayas and colleagues (Zayas et al. 2011)examined whether maternal caregiving at 18 months of age predicts adult attachment to parents, friends, and romantic partners, finding maternal sensitivity is associated with less attachment avoidance and anxiety, while maternal control predicted greater attachment avoidance and anxiety in adult relationships. Adults who are avoidant may distance themselves emotionally from others, displaying anger, mistrust, and coldness in relationships, while those with attachment anxiety may have low self-esteem, clinginess, and a fear of abandonment, often seeking constant reassurance and avoiding social interactions to protect themselves from rejection (Hazan and Shaver 1987, 1994; McNelis and Segrin 2019; Wilson et al. 2013). Attachment anxiety and avoidance form the foundation for many dysfunctional behaviors (Wilson et al. 2013)often leading to relationship dissatisfaction (Finzi et al., 2000), and dissolution in adulthood (Hazan and Shaver 1987, 1994; McNelis and Segrin 2019; Ruiz et al. 2018).

While traditional attachment theory categorized individuals into discrete styles: secure, anxious, avoidant (Bowlby 1969); or, secure, fearful, dismissing, and preoccupied, as later refined by (Bartholomew 1990), more recent research has conceptualized attachment on continuous dimensions of anxiety and avoidance. Fraley and colleagues (Fraley et al. 2000; 2015) developed measures that capture the degree of attachment anxiety and avoidance, allowing researchers to quantify the extent of attachment anxiety or avoidance for a more comprehensive understanding of individual differences. This perspective suggests there are two core underlying dimensions (i.e., anxiety and avoidance) that capture the philosophy of attachment more accurately than four distinct psychological types of attachment (i.e., secure, fearful, dismissing, and preoccupied) to better capture individual differences. In line with this modern approach, the present study measures attachment anxiety and avoidance using a dimensional framework, consistent with the work of Fraley and colleagues (Fraley et al. 2015).

### Attachment Theory and Adverse Childhood Experiences (ACEs)

Adverse Childhood Experiences (ACEs) encompass physical, emotional, or sexual abuse; physical, or emotional neglect; exposure to domestic violence; household substance abuse or poor mental health; parental incarceration; and the separation from, or loss of a caregiver (Felitti et al. 1998). These experiences may lead to constant overactivation of the body’s stress response system, resulting in the prolonged release of stress hormones (e.g., cortisol) that disrupt healthy brain development and may result in long-term damage, with risk increasing as the number of ACEs rises (i.e., a dose-response relationship; (Anda et al. 2005; Clemens et al. 2020).

Attachment theory suggests secure attachment (reflected as low avoidance and low anxiety) emerges when caregivers are sensitive and responsive to their infant’s needs, whereas inconsistent, distant, or rejecting caregiving often leads to insecure attachment patterns (De Wolff & Van Ijzendoorn, 1997). Because many ACEs involve environments where caregivers are harsh, neglectful, or emotionally unavailable, ACEs may serve as an important predictor of attachment insecurity. Empirical evidence supports this link, showing infants exposed to adversity are significantly more likely to develop insecure attachments than infants who were not exposed (Baer and Martinez 2006).

Exposure to ACEs influences attachment patterns that persist into adulthood, whether in relationships with romantic partners, their mothers, or fathers (Zumdahl 2025), with higher cumulative exposure linked to a greater likelihood of insecure attachment and related adverse outcomes (Anda et al. 2005; Barone and Carone 2021; Felitti et al. 1998; Lyons-Ruth 2002; Perry 1999; Scully et al. 2020; Taunton et al. 2021). In a sample of 9,598 individuals (aged 18–100, M = 31), Zumdahl and colleagues(Zumdahl 2025) found higher cumulative ACE scores were positively correlated with attachment anxiety and avoidance across relationship domains (general, mother, father, and romantic). Moreover, Murphy and colleagues (Murphy et al. 2014)found women’s retrospective ACE scores were associated with insecure classifications on the Adult Attachment Interview (i.e., Unresolved/Discordant, reflecting unresolved trauma or loss). The risk of insecure attachment increased gradually with the number of ACEs: 10% for 1 ACE, 17% for 2 ACEs, 38% for 3 ACEs, and 65% for 4 or more ACEs, demonstrating a clear dose-response relationship. Additionally, (Snyder et al. 2024) found a significant indirect effect of ACEs on attachment anxiety in a sample of adults (*n* = 253; 1 ≥ ACEs: 75%; 4 ≥ ACEs: 30%) through increased dissociation and emotion regulation difficulties.

These findings emphasize the importance of early life risk in shaping attachment styles, with adversity experienced during childhood being especially influential.

### Maternal Mental Health: an ACE in its Own Right

The Maternal and Child Health Bureau (2024) defines maternal mental health as the overall emotional, social, and psychological well-being of individuals during and after pregnancy. Common challenges include depression, anxiety, and substance use, which affect mothers across diverse cultural, socioeconomic, and demographic backgrounds (HRSA, 2024). Children of mothers experiencing poor mental health are at increased risk for long-term social, emotional, and behavioral difficulties, including insecure attachment which can persist into adulthood (Anda et al. 2005; Atif et al. 2015; Field et al. 1988). For example, Ruiz and colleagues(Ruiz et al. 2018) found maternal functional impairment (i.e., how severely depression affected their daily functioning) was associated with higher attachment anxiety in offspring during young adulthood.

Poor maternal mental health affects caregiving behaviors in multiple ways. Mothers experiencing depression or anxiety may be emotionally unavailable, less sensitive to cues, or disengaged from their children’s needs (Elgar et al. 2007; Lyons-Ruth 2002). Despite these challenges, children continue to engage with their mothers, even when interactions are marked by negative emotions, misattunement, or failed attempts at reconciliation, as complete avoidance is not a viable option (Barone and Carone 2021; Lyons-Ruth et al. 1999; Tronick and Beeghly 2011). This persistent engagement under strained conditions often contributes to insecure attachment and behavioral difficulties that may persist well into adulthood (Lyons-Ruth 2002; Tupper et al. 2020). Infants of depressed mothers display lower-quality interactions not only with their mothers, but also with strangers, suggesting a generalized depressed response to social interactions (Field 2011; Field et al. 1988).

Maternal mental health is also an adverse childhood experience not uniquely defined by ACEs. Poor maternal mental health has been linked to a greater likelihood of child maltreatment, including physical, sexual, or emotional abuse, or neglect (Plant et al. 2015). Beyond parenting behaviors, maternal mental health challenges may create additional sources of family stress, including marital conflict, substance use, or parental separation, which not only increase children’s emotional and behavioral risk but would add to their adverse childhood experiences (Avison 2010; Hanington et al. 2012; Williams and Cheadle 2016). The standard ACEs questionnaire generally asks whether anyone in the household experienced mental illness, without specifically identifying maternal mental health versus other household members. This distinction is important because maternal mental health difficulties are more common and often more disruptive to child development, given mothers’ typical role as the primary caregiver (Connell and Goodman 2002; Meadows et al. 2007; Noonan et al. 2018). For example, (Kamis 2021; Lyons-Ruth 2002) found among respondents reporting poor parental mental health in childhood, 72.6% reported maternal challenges, compared to 13.1% for fathers. These findings underscore the importance of examining maternal mental health as a distinct construct. Accordingly, the present study conceptualizes maternal mental health separately from general household mental illness to better understand its unique contribution to offspring attachment outcomes, and as a wider context of development.

### The Present Study

A substantial body of research has established links between ACEs and insecure adult attachment. However, prior studies often treat maternal mental health as indistinguishable from the broader ACE construct, given that one of the ten ACE items assesses household mental illness. As a result, little is known about the unique influence of maternal mental health above and beyond the cumulative impact of ACEs. The current study seeks to address this gap by (1) examining the effects of ACEs on adult attachment patterns, with a focus on attachment to participants’ mothers, and (2) testing whether maternal mental health while growing up exerts an additional influence on adult attachment above and beyond ACE exposure.

## Materials and Methods

### Design

Data for this study were collected through a small-town university in the southwest United States via class announcements, social media posts, and word of mouth, using a secure online survey platform. Recruitment took place on campus and in the surrounding community. Prior to survey completion, informed consent was obtained electronically. Participants accessed the survey via a secure, SSL-encrypted URL, which ensured anonymity and data protection. They were informed of their right to skip questions or withdraw from the study at any time without penalty. The study posed minimal risk, with potential discomfort from sensitive questions mitigated by voluntary participation and anonymity. Participants were provided with the contact information of the Principal Investigator (PI) for any questions or concerns. Data were reported only at the group level, stored securely, and accessible exclusively to authorized research personnel to maintain strict confidentiality. Some participants were students, in which case minimal extra credit was provided as compensation; no additional incentives were offered. Information regarding participants’ employment on campus was not collected. The research protocol, including recruitment and data collection procedures, was reviewed and approved by the university’s Institutional Review Board (IRB).

### Participants

The sample comprised 317 participants who were recruited through the university, including the campus and nearby community. The sample was predominantly female (77%) and primarily White (82.3%). Participants’ ages ranged from 18 to 79 (*M* = 32.14, *SD* = 15.10). A significant portion of the participants reported a yearly income of less than $20,000 (30.6%), and the most common educational level was “some college, but no degree” (37.9%). Additionally, 56.6% of participants were single and had never been married. For a detailed breakdown, refer to Table 1.

**Table 1:** Sociodemographic characteristics of participants answering the questionnaires

Characteristics	%	*N*	M	SD
Gender				
Male	22.4	71		
Female	77	244		
Other	0.6	2		
Race				
American Indian or Alaskan Native	0.6	2		
Asian or Asian American	2.2	7		
Black or African American	2.2	7		
Native Hawaii or other Pacific Islander	2.5	8		
White	82.3	261		
Hispanic	7.9	25		
Other (Multiethnic)	2.2	7		
Age			32.14	15.10
Yearly Income				
Less than $20,000	30.6	97		
$20,000 to $34,000	14.3	45		
$35,000 to $49,000	8.8	28		
$50,000 to $74,999	14.3	45		
$75,000 to $99,000	9.1	29		
$100,000+	22.8	73		
Education Level				
Less than high school	0.3	1		
High school diploma or equivalent	7.6	24		
Some college, but no degree	37.8	120		
Associate degree	24	76		
Bachelor’s degree	18	57		
Graduate degree	12.3	39		
Current Relationship Status				
Single	56.6	180		
Married	37.3	118		
Divorced	5.4	17		
Widowed	0.6	2		
ACEs				
0	32.2	102		
1	22.7	72		
2	14.5	46		
3	12.0	38		
4	9.5	30		
5	4.7	15		
6	1.9	6		
7	1.9	6		
8	0.3	1		
9	0.3	1		
10	0.0	0		

*N* = 317

The minimum age of participants was 18, while the maximum was 79

18.6% of participants reported four or more ACEs (≥ 4)

### Measures

#### Adult Attachment to Mother

Adult attachment to the mother was assessed using the Relationship Structures Questionnaire (ECR-RS; (Fraley et al. 2011). The ECR-RS is a 9-item self-report instrument designed to measure two core dimensions of adult attachment: anxiety and avoidance, across four relationship domains, including mothers, fathers, and romantic partners. Participants rated items on a 7-point Likert scale ranging from 1 (*strongly disagree*) to 7 (*strongly agree*). Higher scores on the attachment anxiety subscale indicate greater attachment anxiety, whereas higher scores on the attachment avoidance subscale indicate greater attachment avoidance. Sample items include: “*It helps to turn to this person in times of need*” and “*I often worry that this person doesn’t really care for me.*” Importantly, attachment anxiety and avoidance are conceptualized as continuous dimensions rather than discrete categories. This approach allows for capturing nuanced individual differences (e.g., high anxiety with low avoidance) and aligns with theoretical models in which attachment anxiety reflects monitoring relational availability and attachment avoidance reflects regulation of closeness and dependence (Fraley et al. 2015). Participants in this study responded specifically about their relationship with their mothers. The ECR-RS has demonstrated strong test-retest reliability, with correlation coefficients averaging 0.65 for romantic relationships and 0.80 for parental relationships (Fraley et al. 2011).

#### Adverse Childhood Experiences (ACEs)

Childhood adversity was assessed using the 10-item Adverse Childhood Experiences (ACE) questionnaire developed by Felitti and colleagues (Felitti et al. 1998). This widely used measure evaluates exposure to various forms of childhood adversity, including abuse, neglect, and household dysfunction. Items are scored dichotomously (*0 = no*,* 1 = yes*), with higher total scores indicating exposure to a greater number of adverse experiences. Example items include: “*Before your 18th birthday*,* did a parent or other adult in the household often or very often swear at you*,* insult you*,* put you down*,* or humiliate you?*” and “*Before your 18th birthday*,* did a parent or other adult in the household often or very often push*,* grab*,* slap*,* or throw something at you? Or ever hit you so hard that you had marks or were injured?*” Validation studies demonstrate strong test–retest reliability of the ACE categories (*κ* = 0.75 − 0.86 across domains) and of the total ACE score (weighted *κ*= 0.64; (Dube et al. 2004), supporting its use in retrospective research on childhood adversity and later outcomes.

#### Maternal Mental Health Challenges

Prior to answering questions on maternal mental health, respondents were asked to identify their primary mother figure during childhood. This question did not distinguish whether the primary maternal figure was a biological mother, stepmother, adoptive mother, relative, or non-relative who primarily raised the respondent. Overall maternal mental health was assessed with the question: “*In general*,* how would you describe your mother’s mental health while you were growing up at home?*” Participants responded on a 5-point Likert scale (*1 = Excellent*,* 2 = Very Good*,* 3 = Good*,* 4 = Fair*,* 5 = Poor*). This item was coded such that higher scores indicated poorer maternal mental health. The measure was designed to capture participants’ overall perception of their mother’s mental health, consistent with the use of global single-item assessments in large-scale health surveys (Ahmad et al. 2014); Allen et al. 2022; Lyons-Ruth 2002).

#### Confounding Variables

Participants’ current household income and marital status were included as potential confounding variables. Income was assessed with the question, “*What is your yearly household income?*” with response options ranging from less than *$20*,*000 to $100*,*000* or more. Marital status was assessed with the question, “*Are you married or in an exclusive relationship?*” with response options including *dating exclusively*,* cohabitating*,* married*,* or not in a relationship*. These variables were considered potential confounders because both income and relationship status may influence adult attachment patterns and perceptions of maternal relationships. For example, higher income or being in a supportive, committed relationship may buffer the long-term effects of childhood adversity or poor maternal mental health on adult attachment (Font and Maguire-Jack 2016). Conversely, lower income or lack of a stable relationship could exacerbate the impact of these early experiences. Controlling for these variables allows for a more accurate examination of the associations between childhood maternal mental health, ACEs, and adult attachment with mothers.

#### Statistical Analysis

Analysis proceeded in two main steps. First, we performed bivariate correlations to assess the relationship between maternal mental health, ACEs, and adult attachment to the mother (i.e., anxiety and avoidance). This initial analysis provided a preliminary understanding of the strength and direction of the relationships between key variables (*see* Table 2). Next, we used a two-stage hierarchical multiple regression analysis to test for independent contributions of ACEs and maternal mental health to attachment anxiety and attachment avoidance in the maternal relationship, while controlling for demographic variables (i.e., income and marital status). Income and marital status were assessed in the first step (Model 1), followed by the inclusion of ACEs in the second step (Model 2), and maternal mental health in the third step (Model 3). This approach allowed us to determine how much maternal mental health contributed to explaining attachment anxiety and avoidance, over and above the influence of ACEs, while controlling for potential confounding variables. Specifically, in Model 1, only income and marital status were tested as predictors of both attachment anxiety and avoidance. In Model 2, ACEs were added to examine whether they accounted for additional variance in attachment anxiety and avoidance after controlling for income and marital status. In Model 3, maternal mental health was entered to assess its contribution to explaining attachment anxiety and avoidance after controlling for income, marital status, and ACEs. The regression analyses were performed separately for attachment anxiety and avoidance as the dependent variables (*see* Table 3).

**Table 2:** Means, standard Deviations, and correlations for study variables

Variables	*n*	M	SD	1	2	3	4	5
1. Income	306	^a^	^a^					
2. Marital status	306	^a^	^a^	−0.03				
3. ACEs	317	1.79	1.86	−0.07	−0.03			
4. MMH	317	2.85	1.23	−0.09	−0.07	0.50*		
5. Anxiety	317	1.93	1.62	−0.04	−0.01	0.38*	0.47*	
6. Avoidance	317	2.79	1.73	−0.03	0.00	0.38*	0.52*	0.62*

*p <.01

*MMH* Maternal mental health

^a^ = M and SD are not reported for categorical variables (income, marital status)

**Table 3:** Summary of hierarchical linear regression analyses predicting attachment anxiety and avoidance

Variable	Anxiety			Avoidance		
	B	β	SE	B	β	SE
Model 1						
Income	−0.03	−0.04	0.30	−0.02	−0.03	0.05
Marital status	−0.00	−0.00	0.09	−0.01	−0.01	0.09
Model 2						
Income	−0.01	−0.01	0.04	0.00	0.00	0.05
Marital status	0.01	0.00	0.08	0.00	0.00	0.08
ACEs	0.33	0.38*	0.05	0.35*	0.38*	0.05
Model 3						
Income	0.01	0.02	0.04	0.02	0.03	0.04
Marital status	0.04	0.03	0.07	0.04	0.03	0.07
ACEs	0.17	0.19*	0.05	0.15*	0.17*	0.05
MMH	0.52*	0.39*	0.08	0.61*	0.43*	0.08

*p <.05

*N* = 317

*MMH* Maternal mental health

All statistical analyses were conducted in IBM SPSS Statistics Version 29. Ordinary least squares (OLS) regression was used to estimate direct effects, and model fit was evaluated using R², ΔR², F-tests, and regression coefficients. Assumptions of linearity, homoscedasticity, and multicollinearity were assessed and met. Cases with missing data on the primary variables of interest (ACEs, maternal mental health, and attachment outcomes) were omitted from analyses, while minimal missing data in demographic variables were retained.

## Results

### Descriptives

A total of 317 participants completed the questionnaires, with ages ranging from 18 to 79 (*M* = 32.14, *SD* = 15.10). The sample was predominantly female (77.0%), followed by male (22.4%) and other genders (0.6%). The majority identified as White (82.3%), followed by Hispanic (7.9%), Black or African American (2.2%), Asian or Asian American (2.2%), and other groups. Most participants reported an annual income of less than $20,000 (30.6%) or more than $100,000 (22.8%), and a majority had some college education but no degree (37.8%). In terms of relationship status, a majority were single (56.6%), while 37.3% were married. The number of Adverse Childhood Experiences (ACEs) among the participants varied, with 32.2% reporting zero ACEs, 22.7% reporting one ACE, 14.5% reporting 2 ACEs, 12.0% reporting 3 ACEs, and 18.6% reporting four or more.

### Bivariate Associations between Adult Attachment and Other Risk Factors

Next, we tested for associations between maternal mental health, ACEs, attachment anxiety and avoidance, which ranged from small to moderate in magnitude. Table 2 summarizes these bivariate analyses, displaying the means, standard deviations, and correlations between study variables (i.e., ACEs, maternal mental health, attachment anxiety and avoidance). ACEs were positively correlated with maternal mental health (*r* =.50, *p* <.01), attachment anxiety (*r* =.38, *p* <.01), and attachment avoidance (*r* =.38, *p* <.01). Attachment anxiety and attachment avoidance were strongly correlated with each other (*r* =.62, *p* <.01). Additionally, maternal mental health was positively associated with attachment anxiety (*r* =.47, *p* <.01) and attachment avoidance (*r* =.52, *p* <.01).

### Predicting Adult Attachment To Mother

A two-stage hierarchical multiple regression was conducted to examine predictors of attachment anxiety and avoidance. For attachment anxiety, in Model 1, income and marital status were entered as predictors, but neither significantly predicted attachment anxiety (*β =* −0.04 and *β =* −0.00, respectively, *p* >.05). In Model 2, ACEs were added to the model and contributed significantly to the regression model for attachment anxiety *F*(1, 315) = 52.91, *p* <.001, accounting for 14% of the variance (*β =.*38, *p* <.05). Income and marital status remained non-significant predictors in this model. In Model 3, maternal mental health was added to the regression and, for attachment anxiety specifically, it increased the variance explained to 24% (*β* = 0.39, *p* <.05). This change in *R*^2^ was significant, *F*(1, 314) = 43.65, *p* <.001 (see Table 3). ACEs remained a significant contributor with a reduced effect (*β* = 0.19, *p* <.05), while income (*β =* −0.02, *p* >.05) and marital status (*β =* −0.03, *p* >.05) continued to be non-significant.

For attachment avoidance, in Model 1, income and marital status were entered as predictors, but neither significantly predicted attachment avoidance (*β =* −0.03 and *β =* −0.01, respectively, *p* >.05). In Model 2, ACEs contributed significantly to the regression model for attachment avoidance *F*(2, 316) = 52.24, *p* <.001, also accounting for 14% of the variance (*β =.*38, *p* <.05). Income and marital status remained non-significant predictors in this model. In Model 3, adding maternal mental health increased the variance explained to 29% (*β* = 0.43, *p* <.05), and this change in *R*^2^ was significant, *F*(1, 314) = 65.92, *p* <.001 (see Table 3). ACEs remained a significant contributor with a reduced effect (*β* = 0.17, *p* <.05). Income (*β* = 0.03, *p >*.05) and marital status (*β* = 0.03, *p* <.05) remained non-significant.

The variance in attachment anxiety and avoidance explained by ACEs and maternal mental health is greater than that explained by ACEs alone.

## Discussion

Attachment research has shown childhood adversity, especially when it originates from the primary caregiver, typically the mother, is strongly associated with the development of insecure attachment in childhood, which often persists into adulthood (Zumdahl 2025). While prior research has established a link between childhood adversity and insecure attachment in adulthood, fewer studies have considered whether including the mental health of the primary caregiver, in this study, the mother, adds to the prediction of adult attachment outcomes beyond the effects of childhood adversity alone. Thus, existing cross-sectional studies have not investigated whether the inclusion of maternal mental health explains additional variance in adult attachment outcomes beyond the influence of childhood adversity.

The study results were consistent with our hypothesis, indicating both ACEs and poor maternal mental health are associated with greater attachment anxiety and avoidance in adulthood. When maternal mental health is included to the model, it contributes additional risk, such that individuals exposed to both childhood adversity and poor maternal mental health show greater attachment anxiety and avoidance than those exposed to adversity alone. Once maternal mental health is included to the model (Model 3), the unique effect of ACEs is reduced, suggesting part of their influence may operate through maternal mental health. This pattern suggests some studies focusing solely on ACEs may provide an incomplete picture of their impact if eminent parental factors (e.g., mental health) are not simultaneously considered. In other words, the enduring influence of maternal mental health compounds the risk already posed by childhood adversity, underscoring the importance of accounting for primary caregiver detrimental characteristics when examining long-term attachment outcomes. This is supported by previous findings showing when mothers are mentally unstable and emotionally unavailable, the negative effects of ACEs on attachment anxiety and avoidance are more pronounced (Piché et al. 2011). The aim of this study is not to diminish the significance of ACEs but rather to provide a more nuanced understanding of how vital maternal mental health can exacerbate their impact. Together, these results suggest both childhood adversity and maternal mental health represent meaningful contexts for prevention and intervention efforts (CDC, 2022).

Additionally we found ACEs alone (Model 2) had a similar impact on both attachment anxiety and avoidance. However when maternal mental health was added to the model (Model 3) the combined effect of ACEs and maternal mental health had a stronger impact on attachment avoidance than on attachment anxiety. This finding aligns with Bowlby’s attachment theory which emphasizes specific maternal behaviors during formative years shape distinct attachment styles (e.g. consistently unresponsive caregiving tends to foster avoidant attachment;Bowlby, 1988). Poor maternal mental health is often characterized by emotional withdrawal and reduced sensitivity, conditions that are particularly associated with the development of avoidant attachment (Field et al. 1988). By contrast, attachment anxiety is typically linked to inconsistent responsiveness (i.e., caregiving marked by a mix of attentiveness and unavailability), which may also be influenced by poor maternal mental health, but in a different or less direct way. Thus, the stronger association with avoidance suggests maternal mental health difficulties amplify risks specifically tied to emotional detachment in early caregiving relationships. These early relational patterns have important implications for adult relationships, as the coping mechanisms and less adaptive interpersonal skills formed in interactions with a primary caregiver can persist into adulthood (Field et al. 1988; McNelis and Segrin 2019; Wilson et al. 2013; Zayas et al. 2011). As a result, individuals may display similar reserved or socially withdrawn behaviors in adult relationships, hindering their ability to form close connections.

Taken together, the present findings emphasize maternal mental health may play a unique and meaningful role in shaping adult attachment outcomes, beyond its classification as a single ACE item. Collapsing maternal mental health into a broad measure of ‘household mental illness’ risks overlooking its distinct pathways of influence on children’s lifespan development. While this study did not examine severity or chronicity, the results suggest considering maternal mental health more directly, rather than as one component of childhood adversity.

### Limitations and Future Directions

We recognize several limitations in our study. The primary limitation is the reliance on retrospective self-reports of ACEs and maternal mental health prior to age 18, which may introduce inaccuracies that cannot be objectively verified. Importantly, the measure of maternal mental health represents participants’ subjective recollections of their mothers’ emotional functioning, rather than objective or clinical assessments. This perspective-based approach nevertheless provides valuable insight into how maternal mental health was perceived and internalized by the child, which is itself a meaningful dimension of early relational experience. Earlier research has noted adults may overestimate or underestimate their negative childhood experiences for various reasons, such as current mental health challenges or lack of recall for events occurring before age three (infantile amnesia; (Hardt et al. 2010), with underestimation a more likely occurrence (Hardt and Rutter 2004; Pinto and Maia 2013). However, qualitative studies indicate even very young children can interpret the emotional state of a primary caregiver (Simpson-Adkins and Daiches 2018). With parental difficulties related to maternal mental health (i.e., showing affection, holding, or singing) still being meaningful to the child in the absence of verbal interactions (Beardslee 2004). In addition, Robins and colleagues(Robins et al. 1985) suggest adults, regardless of their own mental health diagnosis, had no systematic bias toward negative recall. Even so, the findings presented here should be interpreted with the understanding no formal test of validity or reliability was possible with this dataset. Future research should address these limitations by incorporating prospective, longitudinal designs that follow families from pregnancy through adulthood, thereby reducing reliance on retrospective reports.

Another limitation of the current study is the use of a single-item, retrospective measure to assess maternal mental health: “*In general*,* would you say your mother’s mental health when you were growing up at home was: Excellent*,* Very Good*,* Good*,* Fair*,* or Poor?*” While single-item measures have historically been criticized for potentially lower reliability and limited capacity for fine-grained assessment (Wanous et al. 1997), recent literature indicates they can be appropriate when the construct is narrow, explicit, and clearly defined (Allen et al. 2022; Bergkvist and Rossiter 2007)and they have the added benefits of reduced participant burden and lower data-processing costs (Allen et al. 2022; Bergkvist and Rossiter 2007). In this study, the question aimed to capture a general construct: the participants’ overall perception of their mother’s mental health, which has been used in previous large-scale surveys (e.g., HILDA Survey; (Watson and Wooden 2021). Nevertheless, it is important to acknowledge reliability, and validity cannot be formally assessed in cross-sectional designs with single items, and subtle dimensions of maternal mental health may not be fully captured. Future research should consider using multi-item measures or repeated assessments to validate and expand upon these findings, particularly for longitudinal studies aiming to find changes in maternal mental health over time.

Although we modeled maternal mental health above and beyond cumulative ACEs, we recognize ACEs are inherently interrelated and some collinearity is inevitable. Moreover, maternal sociodemographic and prenatal information were not available, limiting our ability to account for these influences. Thus, findings should be interpreted within the broader context of childhood adversity.

Another limitation of the present study is the average ACE score in our sample was relatively low, with 32.2% reporting 0 ACEs, 22.7% reporting 1 ACE, 14.5% reporting 2 ACEs, 12.0% reporting 3 ACEs, and 18.6% reporting 4 or more ACEs. As a result, our findings primarily reflect the impact of low-to-moderate ACE exposure and may not generalize to populations experiencing severe childhood adversity. Nonetheless, prior research demonstrates even low levels of ACEs can meaningfully influence outcomes, such as attachment and developmental challenges (Muench et al. 2018; Thomson and Jaque 2017; Webster 2022). These findings support the interpretation measurable effects of adversity can occur at lower levels of ACE exposure. Future research should examine whether the patterns observed here persist or are amplified in populations with higher cumulative adversity.

Sample characteristics limit generalizability. The sample was limited to individuals with access to necessary resources and internet connectivity, which may have excluded participants from lower socioeconomic backgrounds.

Despite these limitations, this study adds to existing literature examining children’s early experiences and their outcomes in adulthood. The current study provided preliminary evidence on how ACEs, combined with poor maternal mental health, influence adult attachment styles. However, it only assessed participants’ attachment to their mothers. We focused on adult attachment with the mother because she is often the earliest and most influential attachment figure. While other caregivers matter, the mother–child attachment in the formative years shapes core expectations of safety and nurturance that persist into adulthood. Studying this relationship offers insight into how early caregiving, especially in the context of maternal mental health, continues to affect attachment patterns across life. Future research should expand to examine other types of attachments, such as those with fathers and romantic partners. For instance, (Zumdahl 2025) found the impact of ACEs on adult attachment differed depending on the attachment figure. Specifically, for parental attachments (both mothers and fathers), the timing of ACEs significantly influenced adult attachment, whereas attachment to romantic partners was not affected by the timing of childhood adversities.

### Implications

Our study shows both ACEs and poor maternal mental health are associated with attachment anxiety and avoidance later in life. The most important finding of our study is the combined effect of ACEs along with poor maternal mental health, which significantly increases the variance explained for attachment anxiety and avoidance later in life. These findings have implications for how we support mothers of young children. They suggest it is important to focus on improving maternal mental health in addition to protecting children from adversity to prevent insecure attachment. Addressing mothers’ mental health needs during their children’s early years may significantly shape the well-being and development of the children as they grow into adults. While we may not be able to protect all children from adverse experiences, we can help mothers address their mental health needs and provide the emotional support their children need to navigate those challenges. Raising awareness of maternal mental health and the mother-child relationship can drive a shift in medical and therapeutic systems from reactive approaches to preventative ones, enhancing the long-term well-being of both mothers and children.

## Appendix 1

**Table:** 

Adverse Childhood Experiences
● Question: Before your 18th birthday, did a parent or other adult in the household often or very often… ● Answers: 1 = Yes, 0 = No
1. Swear at you, insult you, put you down, or humiliate you? *Or act* in a way that made you afraid that you might be physically hurt?
2. Push, grab, slap, or throw something at you? Or ever hit you so hard that you had marks or were injured?
3. Touch or fondle you or have you touched their body in a sexual way? *Or attempt* or actually have oral, anal, or vaginal intercourse with you?
4. Feel that no one in your family loved you or thought you were important or special? *Or* feel that your family didn’t look out for each other, feel close to each other, or support each other?
5. Feel that you didn’t have enough to eat, had to wear dirty clothes, and had no one to protect you? *Or feel* that your parents were too drunk or high to take care of you or take you to the doctor if you needed it?
6. Before your 18th birthday, was a biological parent ever lost to you through divorce, abandonment, or other reason?
7. Before your 18th birthday, was your mother or stepmother: often or very often pushed, grabbed, slapped, or had something thrown at her? *Or* sometimes, often, or very often kicked, bitten, hit with a fist, or hit with something hard? *or* ever repeatedly hit over at least a few minutes or threatened with a gun or knife?
8. Before your 18th birthday, did you live with anyone who was a problem drinker or alcoholic, or who used street drugs?
9. Before your 18th birthday, was a household member depressed or mentally ill, or did a household member attempt suicide?
10. Before your 18th birthday, did a household member go to prison?

## Appendix 2

**Table:** 

Attachment
Requirements: ● Please answer the following questions about your dating or marital partner. Note: If you are not currently in a dating or marital relationship with someone, answer these questions with respect to a former partner or a relationship you might have. ● Please answer the following questions about your mother (or mother-like figure)/father (or father-like figure)
Response options: 1 = *strongly disagree*, 2 = *moderately disagree*, 3 = *slightly disagree*, 4 = *neither disagree or agree*, 5 = *slightly agree*, 6 = *moderately agree*, 7 = *strongly agree*
Questions:
1. It helps to turn to this person in times of need.
2. I usually discuss my problems and concerns with this person.
3. I talk things over with this person.
4. I find it easy to depend on this person.
5. I don’t feel comfortable opening up to this person.
6. I prefer not to show this person how I feel deep down.
7. I often worry that this person doesn’t really care for me.
8. I’m afraid that this person may abandon me.
9. I worry that this person won’t care about me as much as I care about him or her.

## Appendix 3

**Table:** 

Maternal Mental Health
Instructions: Please answer the following question about your mother
Response options: 1 = *Excellent*, 2 = *Very good*, 3 = *Good*, 4 = *Fair*, 5 = *Poor*
Question: In general, would you say your mother’s mental health when you were growing up at home was: Excellent, Very Good, Good, Fair or Poor?
